# The Molecular Chaperone Artemin Efficiently Blocks Fibrillization of TAU Protein *In Vitro*

**DOI:** 10.22074/cellj.2018.4510

**Published:** 2017-11-04

**Authors:** Zahra Khosravi, Mohammad Ali Nasiri Khalili, Sharif Moradi, Reza Hassan Sajedi, Mehdi Zeinoddini

**Affiliations:** 1 Department of Biosciences and Biotechnology, Malek Ashtar University of Technology, Tehran, Iran; 2 Department of Stem Cells and Developmental Biology, Cell Science Research Center, Royan Institute for Stem Cell Biology and Technology, ACECR, Tehran, Iran; 3 Department of Developmental Biology, University of Science and Culture, Tehran, Iran; 4 Department of Biochemistry, Faculty of Biological Sciences, Tarbiat Modares University, Tehran, Iran

**Keywords:** Aggregation, Alzheimer’s Disease, Artemin, Chaperone, TAU Protein

## Abstract

**Objective:**

Aggregation of the TAU proteins in the form of neurofibrillary tangles (NFTs) in the brain is a common risk factor in tauopathies including Alzheimer’s disease (AD). Several strategies have been implemented to target NFTs, among which chaperones, which facilitate the proper folding of proteins, appear to hold great promise in effectively inhibiting TAU polymerization. The aim of this study was to analyze the impact of the chaperone Artemin on TAU aggregation *in vitro*.

**Materials and Methods:**

In this experimental study, recombinant TAU- or Artemin proteins were expressed in E.coli bacteria, and purified using ion-exchange and affinity chromatography. Sodium dodecyl sulfate-poly acrylamide gel electrophoresis (SDS-PAGE) was used to run the extracted proteins and check their purity. Heparin was used as an aggregation inducer. The interaction kinetics of TAU aggregation and disassembly was performed using thioflavin T (ThT) fluorescence analysis and circular dichroism (CD) spectroscopy.

**Results:**

Ion-exchange and affinity chromatography yielded highly pure TAU and Artemin proteins for subsequent analyses. In addition, we found that heparin efficiently induced TAU fibrillization 48 hours post-incubation, as evidenced by ThT assay. Importantly, Artemin was observed to effectively block the aggregation of both physiologic- and supra- physiologic TAU concentrations in a dose-dependent manner, as judged by ThT and CD spectroscopy analyses.

**Conclusion:**

Our collective results show, for the first time, that the chaperone Artemin could significantly inhibit aggregation of the TAU proteins in a dose-dependent manner, and support Artemin as a potential potent blocker of TAU aggregation in people with AD.

## Introduction

Alzheimer’s disease (AD) is the best-known of the tauopathies which are characterized by the aggregation of TAU proteins in the brain (1, 2). In AD, TAU aggregates are observed in the form of intracellular neurofibrillary tangles (NFTs) (3, 4). NFTs are formed following the hyperphosphorylation and/or overexpression of TAU protein. Highly expressed in neurons, TAU protein is a microtubule-associated protein which facilitates microtubule-mediated trafficking within cells (5, 6). Increased intracellular concentrations and subsequent aggregation of TAU protein can reportedly impair axonal transportation of the organelles, particularly mitochondria (7-10). AD is diagnosed by short-term memory loss and gets worse over time, causing patients to suffer from additional symptoms including significantly decreased motivation, behavioral problems, and sleep disturbance (11-13). These symptoms apparently emerge because NFTs promote neuronal damage and death, synaptic dysfunction, and ultimately brain shrinkage and patient death (14-16). It is therefore important to develop strategies to target NFTs in AD patients (3, 17, 18). Since TAU aggregation in neurons could be, at least partly, attributed to abnormal folding (19, 20), agents (e.g. small molecules, proteins, or other factors) which inhibit the misfolding of proteins and/or promote proper protein folding might be applicable in this setting (21, 22). In fact, it has been reported that methylthioninium chloride (22), flavonoids (23), rhodanines (24), gossypetin (25), polyphenolic compounds (23), R55 (26), and N744 (27) could promote the disassembly of protein aggregates *in vitro*. Additionally, other factors (e.g. methylene blue) inhibit TAU fibrillization not only *in vitro* but also in animal models (28, 29).

Chaperones promote the proper folding of proteins as well as inhibit protein misfolding. They convert misfolded proteins into modified protein structures that are detectable by the ubiquitin-proteasome system (UPS) for degradation, providing an efficient clearance system for cells to get rid of the potential toxicity caused by misfolded proteins (30). Therefore, chaperones may have the ability to be used as potential inhibitors of TAU polymerization (31). Artemin is a heat-resistant protein and constitutes 12-13% of proteins within the cysts of the aquatic crustacean *Artemia*. It protects *Artemia* cysts from stressful conditions through its different features, including high hydrodynamic hydration and its ability to renature aggregated and denatured protein structures (32, 33). Artemin has, for example, been reported to inhibit the heat-induced aggregation of the protein citrate synthase *in vitro* (34). In addition, ‘denatured’ carbonic anhydrase and horse-radish peroxidase are restored to their normal folding and function in the presence of Artemin, indicating that Artemin is a potent chaperone capable of blocking aggregation and denaturation of these proteins (35).

In the present study, we tested whether Artemin protein from *Artemia urmiana* can inhibit TAU aggregation in vitro. We found that Artemin was able to efficiently block TAU fibrillization in a dose-dependent manner. We thus indicate for the first time that AD-associated TAU aggregates could be disintegrated through the use of Artemin *in vitro*. Our results might be applicable to target TAU aggregates in cell-based as well as animal models of TAU aggregation, providing a new avenue for chaperone research on tauopathies.

## Materials and Methods

### Expression and purification of TAU and Artemin

In this experimental study, in order to ectopically express recombinant human TAU protein in bacteria, a His-tagged TAU DNA (1N4R) (Eurofins MWG Operon Company, USA) was cloned into isopropyl-beta-Dthiogalactopyranoside (IPTG)-inducible pET-21a (+) expression vector, and the resulting vector was transformed into E.coli BL21 (DE3) bacteria for expression. Vectortransformed bacteria were incubated and grown at 37˚C in 1 L Luria Broth containing 100 μg/ml ampicillin at 200 rpm to an optical density of 0.6 at 600 nm. IPTG was used at a final concentration of 1 mM to induce TAU expression in the bacteria. After 4 hours incubation at 37˚C, bacteria were harvested and resuspended in lysis buffer (1 mM EDTA, 5 mM DTT, 50 mM NaCl, 20 mM Tris-HCl, 0.1 mM PMSF (all from Sigma-Aldrich, USA, pH=7.5) and were then broken by sonication on ice (Soniprep 150). The supernatants were filtered, loaded onto a SP Sepharose column, and washed with lysis buffer. A linear gradient of salt (0.05-1 M NaCl) in the same buffer without DTT and EDTA was used to elute the TAU protein. Next, we used a Nickel-Chelating Sepharose column (pre-equilibrated with 20 mM Tris-HCl, 0.5 M NaCl, 10 mM Imidazole (Sigma-Aldrich, USA), and 0.3% Triton X-100 (Merck, USA, pH=7.5) to load the fractions containing TAU protein. Then, a solution consisting of 20 mM Imidazole and 20 mM Tris-HCl (pH=7.5) was used to wash out the unbound proteins, and the TAU-containing fractions were eluted by a linear gradient of Imidazole (20-600 mM).

To misexpress Artemin in bacteria, a His-tagged Artemin-encoding pET-28a/Art expression vector with Kanamycin resistance (Novagen) was used to transform *E.coli* BL21 DE3 bacteria. The same procedures and steps as for TAU expression and purification (described above) were performed to express and purify Atermin, except that i. Kanamycin (50 μg/ml, Qiagen, Germany) was used for antibiotic selection of the vector-containing bacteria; ii. Lysis buffer consisted of 50 mM NaH2PO4 (Merck, USA), 300 mM NaCl, 1 mM PMSF, pH=8.0, and iii. Only affinity chromatography was used to purify Artemin, using washing buffer (50 mM NaH_2_PO_4_, 300 mM NaCl, and 10 mM Imidazole, pH=8.0) and then elution buffer (50 mM NaH2PO4, 300 mM NaCl, and 250 mM Imidazole, pH=8.0). Bradford assay and Coomassie Brilliant Blue staining of 10% SDS-PAGE were used to estimate protein concentration and purity, respectively. The purified proteins were exchanged into 10 mM PBS buffer pH=7.4 (Thermo Fisher Scientific, USA) and transferred to -70˚C for long-term storage. This work was approved by the Scientific and Ethics Committee of Malek-Ashtar University of Technology.

### Induction of TAU aggregation by heparin

Polymerization of TAU was induced in the presence of the aggregation inducer heparin (Sigma-Aldrich, USA) with a TAU: heparin molar ratio of 4:1. The kinetics of TAU aggregation in the presence of heparin was performed with physiologic- (4 μM) and supra-physiologic (20 μM) concentrations of TAU (36-38) for 0, 12, 24, 36, 48, and 72 hours of incubation at 350 rpm and 37˚C (final volume of the reaction mixture was 200 μl). As a reducing agent, 40 μM DTT buffer (pH=7.4) was prepared in Tris-HCl and used during the TAU-heparin incubations.

### Thioflavin T fluorescence analysis

The interaction of aggregated TAU with the amyloid marker ThT (Sigma-Aldrich, USA) was analyzed by fluorescence measurements as described below. A fresh ThT stock solution was prepared in double-distilled water according to the vendor’s instructions (Sigma-Aldrich, USA), and filtered (0.22 μm pore size) before use to remove insoluble particles. Recombinant TAU (4 μM and 20 μM) was incubated in 10 mM HEPES buffer (pH=7.4) containing 5 mM DTT, 100 mM NaCl, and 20 μM ThT (Sigma-Aldrich, USA) at 350 rpm and 37˚C for 48 hours in the presence of heparin. The resulting 200-μl solutions, which were run in triplicate, were added to separate wells of a 96-well plate in a CYTATION/3 imaging microplate reader (Biotek, USA) with an excitation wavelength of 440 nm and emission wavelength of 450-550 nm.

### Circular dichroism spectroscopy

The secondary and tertiary structures of the proteins were assessed by CD spectroscopy. TAU protein was mixed with heparin in 10 mM HEPES buffer (Sigma-Aldrich, USA, 100 mM NaCl, 5 mM DTT, pH=7.4) and incubated at 350 rpm and 37˚C for 48 hours. A 1.0-mmpath- length quartz cell (Helma, Germany) was used for measurements in the far-UV region. All the CD spectra were recorded over the range of 190-250 nm at 37˚C. Measurements of the CD spectra were performed using an Aviv Model 215 Spectropolarimeter (Lakewood, USA). For each sample, eight scans, each with a speed of 50 nm/ minute, were performed and averaged, and the resulting spectra were normalized to the blank. The percentages of TAU protein secondary structures in the absence or presence of heparin and Artemin were estimated on a JASCO J-715 CD spectropolarimeter (Jasco Inc, Japan).

### Sodium dodecyl sulfate-poly acrylamide gel electrophoresis

The aggregated TAU was mixed with SDS sample buffer (50% stacking gel buffer, 5% SDS, 5% β-mercaptoethanol, 5% bromophenol blue, 0.25% glycerol, pH=6.8, all from Sigma-Aldrich, USA) and heated to 100˚C for 5 minutes. The samples were run on SDS-PAGE gels containing 10% polyacrylamide.

### Statistical analysis

Data were analyzed with Student’s t test using GraphPad PRISM™ software. A P<0.05 was considered statistically significant.

## Results

### Expression and purification of recombinant human TAU protein

**Fig.1:**
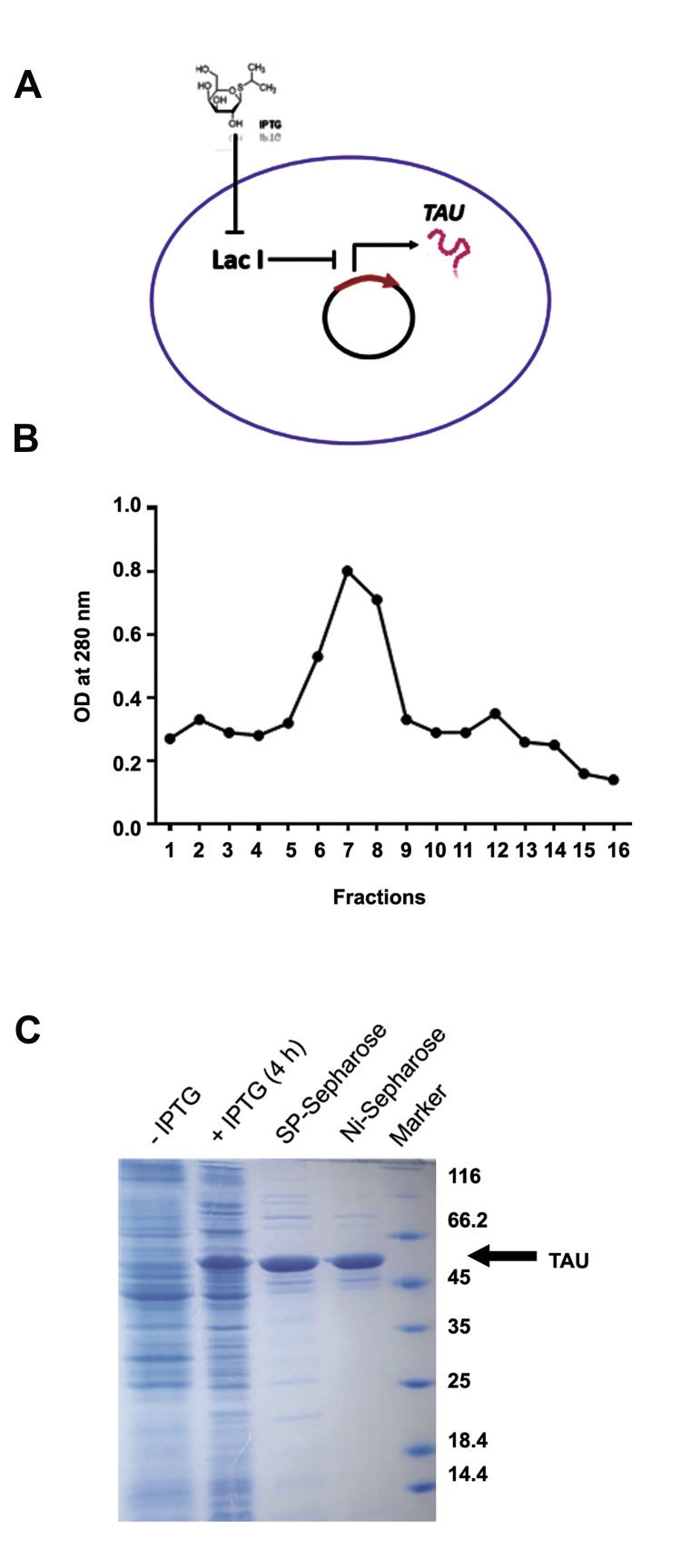
Expression and purification of recombinant TAU. A. Schematic showing the induction of TAU expression in bacteria by IPTG, B. Elution fractions of chromatography for TAU purification, and C. SDS-PAGE indicating the induction of TAU expression by IPTG and its purification by chromatography. IPTG; Isopropyl-beta-D-thiogalactopyranoside, SDS-PAGE; Sodium dodecyl sulfate-poly acrylamide gel electrophoresis, and OD; Optical density.

Recombinant TAU protein provides a suitable *in vitro* model to evaluate TAU protein aggregation. To prepare recombinant TAU protein, we used an IPTG-inducible pET-21a (+) expression vector to misexpress recombinant TAU in *E.coli* bacteria (Fig .1A). Total protein was extracted from bacteria which had been treated for 3-4 hours with IPTG, followed by recombinant TAU purification using ion-exchange and affinity chromatography, respectively. Multiple elution fractions were harvested from the chromatography columns, whose optical density, as determined using a spectrophotometer, indicated that the most concentrated elution fractions were those eluted in the middle of the purification procedure (Fig .1B). These highly concentrated TAU-rich fractions were collected for further analysis. Next, we used SDS-PAGE to visualize the quality and purity of the purified TAU proteins following chromatography. Results of the SDS-PAGE analysis (Fig .1C) indicated that, in contrast to the vector-containing bacteria not induced by IPTG (Fig .1C, IPTG lane), treatment with IPTG led to the emergence of a very thick protein band in the size range for human TAU protein (~50 kDa) within the extracted proteome of the bacteria (Fig .1C, +IPTG lane). Importantly, purification of the recombinant TAU protein was observed to be much higher after affinity chromatography with SPsepharose columns (Fig .1C, SP-sepharose lane). An even higher purification of recombinant TAU protein was obtained when the output of the affinity chromatography was subjected to ion-exchange chromatography with Ni-sepharose columns (Fig .1C, Ni-sepharose lane). These results show that we have successfully been able to induce the expression of recombinant human TAU protein in bacteria and obtain a highly purified TAU protein *in vitro* for subsequent analyses.

### Heparin efficiently induced TAU aggregation *in vitro*

#### Analysis of TAU aggregation induced by heparin using ThT assay

Because we wanted to analyze the effect of the molecular chaperone Artemin on the aggregation of TAU protein, we needed to provide an in vitro model of TAU aggregation, and to this end, we used an efficient inducer of TAU fibrillization. Several agents have been found to promote TAU aggregation (27, 39), among which we chose the extracellular matrix component heparin as a well-established aggregation inducer (40-42). To optimize and determine the length of time required for heparin treatment to induce TAU aggregation, we investigated the kinetics of aggregation of the physiologic concentration of TAU (4 μM) in the presence of heparin using the ThT assay.

**Fig.2:**
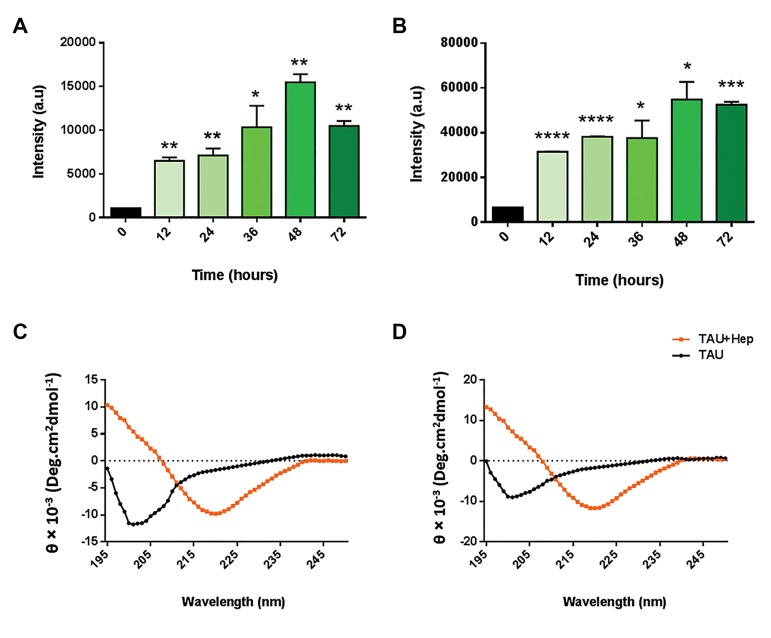
Induction of TAU aggregation using heparin. Optimization of TAU aggregation induction using heparin. A. Barplot showing the ThT analysis of heparin-induced aggregation of physiologic (4 μM) TAU, B. Barplot showing the ThT analysis of heparin-induced aggregation of supra-physiologic (20 μM) TAU. CD spectroscopy analysis indicating the induction of C. TAU aggregation by heparin for physiologic- and D. Supra-physiologic TAU.

Results of the ThT assay (Fig .2A) indicated that heparin promoted the induction and acceleration of TAU aggregation in a time-dependent manner, meaning that TAU aggregation increased in an almost linear manner over time, although TAU aggregation induced by heparin started decreasing 72 hours post-treatment. This finding shows that the most significant induction of physiologic TAU aggregation by heparin occurs 48 hours posttreatment. Additionally, we performed the same procedure with a supra-physiologic TAU concentration (20 μM) to examine whether the starting TAU concentration might affect the kinetics of TAU aggregation. We found that, as with physiologic TAU concentration, heparin induced the efficient fibrillization of supra-physiologic TAU in a time-dependent manner, and that the aggregation of TAU was slightly, but not significantly, decreased 72 hours after heparin addition (Fig .2B). Not surprisingly, heparin was able to induce a much higher aggregation of TAU proteins when the supra-physiologic concentration was used (Fig .1A, B). This indicates that heparin can not only successfully induce TAU fibrillization, but is also able to accelerate TAU aggregation in a time- and TAU-concentration-dependent manner. In this way, we established optimal conditions for the induction of TAU aggregation by heparin.

#### Analysis of TAU aggregation induced by heparin using CD spectroscopy

Next, to examine structural changes in TAU in the presence of heparin in a more accurate manner, we used CD spectroscopy. The CD assay works on the basis of detecting structural and conformational changes in the protein of interest in a changing environment. To analyze the structural changes in TAU protein induced by heparin, we performed CD spectroscopy at 195- 260 nm (i.e. far UV) in a 1 mm quartz cell. The CD spectrum of the far UV region is very sensitive to small changes in the secondary structure of proteins, while the near UV region indicates changes in the tertiary structure. Since TAU does not have a tertiary structure, we used the CD spectrum in the far UV region. The CD spectrum of monomeric TAU (i.e. in the absence of heparin) normally exhibits the structural pattern of a random coil.

Our results indicated that before heparin treatment, TAU displayed a high degree of negative ellipticity at 200 nm, which is indicative of the presence of a random coil structure that does not have an absorbance spectrum at 218 nm. In contrast, we found that when monomeric TAU is treated with heparin for 48 hours, an absorbance spectrum could be observed at 218 nm both for physiologic- (Fig .2C) and supra-physiologic TAU (Fig .2D), meaning that TAU had been successfully aggregated by heparin. Taken together, we were able to confirm the results of the ThT assay by CD spectroscopy and demonstrate that heparin was able to efficiently induce the formation of aggregated TAU *in vitro*.

#### Artemin efficiently inhibits TAU protein aggregation

**Fig.3:**
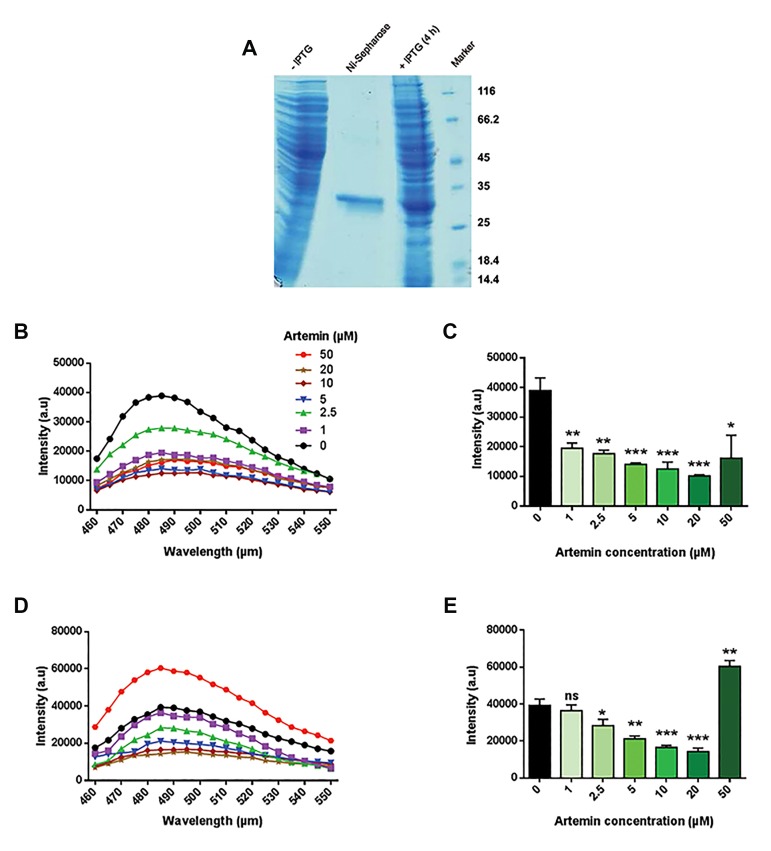
Effect of Artemin on heparin-induced TAU aggregation. A. SDS-PAGE analysis showing the induction of Artemin expression by IPTG and its purification by chromatography, B. ThT spectra of Artemin treatment of heparin-induced aggregation of physiologic (4 μM) TAU, C. Simplified barplot representation of ThT data indicated in B, D. ThT spectra of Artemin treatment of heparin-induced aggregation of supra-physiologic (20 μM) TAU, and E. Simplified barplot representation of ThT data indicated in D. IPTG; Isopropyl-beta-D-thiogalactopyranoside and SDS-PAGE; Sodium dodecyl sulfate-poly acrylamide gel electrophoresis.

To analyze the effect of Artemin on TAU aggregates, we firstly ectopically expressed recombinant Artemin in the *E.coli* strain *BL21 DE3* using an IPTG-inducible pET-28a/Art vector, and extracted total protein 3-4 hours post IPTG treatment (33, 35). To purify Artemin, we then used a Ni-NTA agarose column only (i.e. we did not perform ion-exchange chromatography to further purify it, as the Ni-NTA agarose column yielded a highly purified band corresponding to the size of the native Artemin) (Fig .3A). Next, we examined the effect of different concentrations of Artemin (1 μM, 2.5 μM, 5 μM, 10 μM, 20 μM, and 50 μM) on the efficiency of heparin-induced TAU fibrillization. Interestingly, we observed that treatment of heparin-aggregated physiologic TAU with different concentrations of Artemin led to the efficient inhibition of TAU fibrillization 48 hours post-treatment, as evidenced by ThT assay (Fig .3B, C). Importantly, we found that Artemin effectively diminished the formation of TAU aggregates in a dose-dependent manner, although we observed strong inhibition of TAU aggregation even at 1 μM Artemin. This observation indicated that even low concentrations of Artemin were able to block TAU fibrillization, although the most potent inhibitions were observed to be exerted by higher Artemin concentrations (up to 20 μM), which is indicative of the significant potency of this chaperone in inhibiting aggregated TAU. More importantly, not only did Artemin significantly reduce the rate of 4 μM TAU fibrillization, but it also markedly inhibited the heparin-induced aggregation of supra-physiologic TAU (20 μM) in a dose-dependent manner, as judged by ThT assay (Fig .3D, E). This result indicates that the different starting concentrations of the substrate (i.e. TAU) before induction of TAU aggregation does not significantly interfere with the capability of different concentrations of Artemin (1, 2/5, 5, 10, and 20 μM) to inhibit the formation of TAU aggregates. Unexpectedly, we observed that the strong inhibitory effect of Artemin on TAU aggregation was reversed when a very high concentration of Artemin (50 μM) was added (Fig .3D, E). It may be that very high concentrations of Artemin interfere with its ability to effectively access aggregated TAU. Another possibility is that because a higher concentration of heparin was used to induce supra-physiologic TAU aggregation, heparin might have caused the Artemin protein to aggregate as well, leading to the high aggregation rate observed. Additionally, some aggregation inhibitors have also previously been reported to induce aggregation at high concentrations (43), which may also be the case with Artemin.

**Fig.4:**
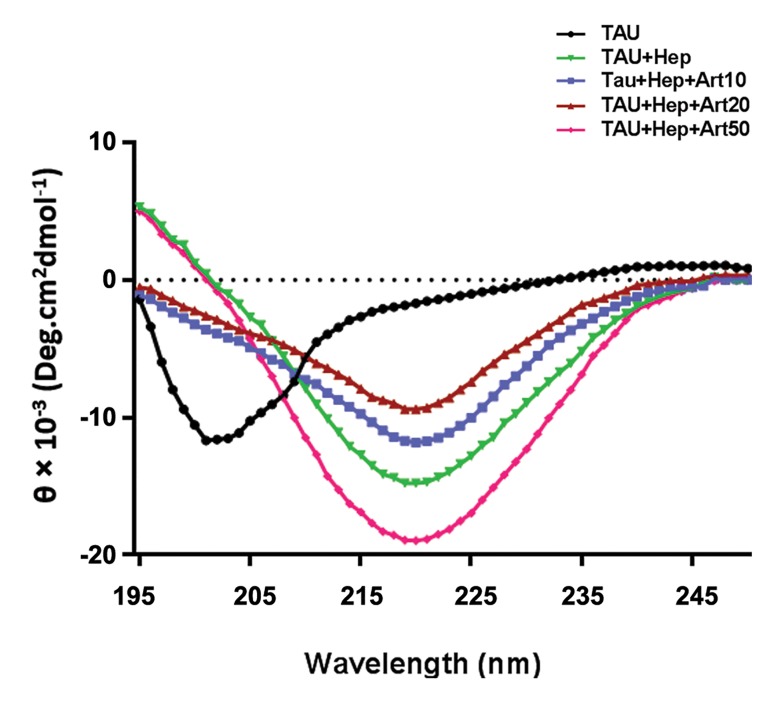
Circular dichroism (CD) analysis indicating the effect of different concentrations of Artemin (10, 20, and 50 μM) on heparin-induced TAU aggregation.

Next, CD spectroscopy was performed to evaluate the conformational changes of TAU induced by heparin, thereby confirming the results of ThT assay. We observed that prior to heparin treatment, there was a high degree of negative ellipticity at 200 nm, which is due to the existence of a random coil structure that lacks an absorbance spectrum at 218 nm (Fig .4). After heparin treatment, we could detect an absorbance spectrum at 218 nm, indicating successful aggregation of TAU. Importantly, upon Artemin treatment, we found that the aggregated TAU exhibited a marked reduction in β-sheet structure and an increase in disordered structures (β-turn and unstructured random coils) (Table 1), as evidenced by the increased negative band at 200 nm as well as a decreased absorbance spectrum at 218 nm (Fig .4), which is indicative of disassembly of the β-sheet structure by Artemin. Of note, high concentrations of Artemin (50 μM) were observed to induce a high level of TAU aggregation (Fig .4, Table 1) which is consistent with the ThT results. In summary, we conclude that the chaperone Artemin is capable of inhibiting TAU aggregation in an effective manner.

**Table 1:** Calculated percentages of TAU protein structure based on CD spectra


	TAU	TAU+Heparin	TAU+Heparin+Art10	TAU+Heparin+Art20	TAU+Heparin+Art50

α-helix	2	8	3	6	2
β-sheet	5	68	53	46	83
β-turn	21	14	21	27	5
Random coil	72	10	23	21	10


## Discussion

In the present study, we investigated the potential of a heat-resistant chaperone protein known as Artemin (33, 35) to block the aggregation of TAU proteins *in vitro*. To this end, recombinant TAU and Artemin proteins were separately misexpressed in bacteria using IPTGinducible vectors coding for TAU and Artemin proteins, respectively. Notably, we utilized the ability of heparin as an inducer to promote the formation of TAU fibrils from physiologic and supra-physiologic concentrations of monomeric TAU protein in a cell-free model system. In fact, heparin has previously been used by other groups to effectively promote the aggregation of TAU protein in vitro (25, 27, 39). Using several techniques, we confirmed that heparin could indeed induce the formation of TAU aggregates in an efficient manner.

We then sought to determine the impact of Artemin on TAU protein aggregation. Interestingly, we found using CD spectroscopy and ThT assay that Artemin inhibited the fibrillization of TAU protein in a dose-dependent manner. Artemin’s inhibitory effect was observed not only at physiologic- but also at supra-physiologic concentrations of TAU protein, which suggests that Artemin is a robust inhibitor of TAU aggregation. To the best of our knowledge, this is the first time that Artemin (in our case, isolated from *Artemia urmiana*) is reported to inhibit the aggregation of TAU as an AD-associated protein. This suggests that Artemin might be a suitable candidate for the treatment of tauopathies, in particular AD, but only after it has been rigorously investigated using cell-based models of AD. Notably, neural cells derived through stem cell differentiation and/or cell fate reprogramming technologies (44-46) have the potential to provide proper neural cell sources needed for in vitro cell-based assessment of TAU aggregation. Animal models of TAU aggregation (28, 29) would be the next step to explore the impact of Artemin *in vivo*. The ability of Artemin to disassemble TAU fibrils is consistent with previous findings reporting that Artemin could significantly inhibit the heat-induced aggregation of citrate synthase (35). It has also been observed to effectively restore the native structure and the normal function of denatured carbonic anhydrase and horse-radish peroxidase (33, 35). These findings indicate that Artemin has the potential to restore the native structure and therefore normal function of proteins which have been exposed to denaturing or aggregating conditions. Taken together, these findings indicate that Artemin is a potent inhibitor of TAU polymerization *in vitro*.

Artemin may exert its inhibitory effect on TAU fibrillization through certain motifs on its surface, since specific motifs mediate the major functions of a protein (47, 48). It might be interesting to examine the effect of short peptide motifs derived from the Artemin protein on the aggregation kinetics of TAU protein. These analyses may bring the potential clinical use of Artemin and/ or Artemin-derived peptides in patients with AD a step closer, provided that Artemin and/or its derivatives are able to inhibit TAU aggregation in cell-based assays as well as in animal models of TAU aggregation.

## Conclusion

Targeting TAU aggregates, which are a major risk factor in tauopathies, including AD, is of critical importance. Several strategies have been used to target TAU fibrils. Among these, the role of chaperones as key players within the cells has recently drawn the attention of researchers seeking a way to inhibit pathologic protein aggregation. In the present study, we demonstrated that the chaperone Artemin was able to block the polymerization of TAU proteins in a dose-dependent manner in a cell-free model system. Artemin has previously been observed to inhibit not only the aggregation of the enzymes horseradish peroxidase and carbonic anhydrase, but also the denaturation of the enzyme citrate kinase. In this study we show that it also inhibits TAU aggregation, suggesting that Artemin might have an intrinsic capability to inhibit the misfolding, aggregation and denaturation of different proteins, including the TAU protein. The next step of interest in this research would be to see whether Artemin is also able to inhibit TAU aggregation *in vivo*.
